# Supplementary material for: Gamma-band synchronization between neurons in the visual cortex is causal for effective information processing and behavior
Source: Nat Commun. 2025 Aug 11;16:7380. doi: 10.1038/s41467-025-62732-8 (PMC12340064; doi:10.1038/s41467-025-62732-8)
Supplement: Supplementary file 1 — Supplementary Information [file 41467_2025_62732_MOESM1_ESM.pdf]

Supplementary information

Gamma-Band Synchronization between Neurons in the visual cortex is causal for effective Information processing and Behavior

Eric Drebitz[1]*, **Lukas-Paul Rausch**[1], **Andreas K. Kreiter**[1]

[1]Cognitive Neurophysiology, Brain Research Institute, University of Bremen, Bremen, Germany

*Corresponding author. Email: drebitz@brain.uni-bremen.de

Supplementary Notes 1-4 2-4
Supplementary Figures 1-9 5-13
Supplementary Table 1 14

Supplementary Notes 1: Comparison of V4 LFP-power spectra

To assess the similarity of γ-LFP characteristics during the two task conditions with two stimuli in the V4 RFs and either one of them attended, we compared the average power spectra calculated for the MCs 2/3 (Fig. 1C). For the γ-peak frequencies in V4, we observed no significant differences when the area V2 population receiving ICMs responded to the attended stimulus (target stim., monkey B: mean = 83 Hz ± 4.3 Hz SD; monkey T: mean = 72 Hz ± 8.5 Hz SD) or the non-attended stimulus (distractor stim., monkey B: mean = 81 Hz ± 4.0 Hz SD, $p = 0.0802$, $t = 1.811$; monkey T: mean = 77 Hz ± 15.8 Hz SD, $p = 0.2458$, $t = 1.1789$; all paired sample *t*-test, two-sided). Supplementary Figure 2 illustrates these power spectra, representing averages across the power spectra calculated for the V4 sites included in the analysis of RT-modulations (Fig. 2C). The latter were derived from trials without ICM (Fig. 1C). For detailed information on the spectral decomposition and power calculation, please refer to Drebitz et al., 2018. Normalization of each site's power spectrum was performed by subtracting the power spectrum of the corresponding baseline period (Fig. 1C) and dividing it by the baseline power spectrum.

Supplementary Notes 2: Comparison of RT-modulation between attentional conditions

To test whether the pooling of data across attentional conditions is legitimate, we compared the effect sizes induced by ICM on RT between these two conditions (SFig. 3). V2 neurons receiving ICM either processed the relevant target stimulus or the irrelevant distractor stimulus (while in the V4 RF, there was always one attended stimulus). To compare differences between the impact of ICM-evoked spikes arriving during the effective or the ineffective γ-phase range of the V4-population, both phase ranges were determined separately for each attentional condition of each animal (± 45° around maximum and minimum as in Fig. 2C). This data split made it necessary to pool RTs of both animals for each attentional condition and phase range. We observed that RTs of both attentional conditions falling into the effective phase range were, on average, significantly longer (median target stim: 61 ms, n = 94; median distractor stim: 74 ms n = 75) than during the ineffective phase ranges (median target stim: 4 ms, n = 85, $p = 7.6348*10^{-4}$, $z = 3.7308$; median distractor stim: 11 ms, n = 111, $p = 1.4155*10^{-5}$, $z = 4.6368$, all comparisons Wilcoxon rank-sum tests, two-sided). These results confirm a similar and phase-dependent effect of ICMs on RTs for both conditions. In addition, there was neither a significant difference between RTs of the effective phase ranges of both conditions ($p = 0.1382$, $z = 1.4826$,) nor between RTs of the ineffective phase

ranges ($p = 0.4338$, $z = 0.7826$; all statistical comparisons: Wilcoxon rank-sum tests, two-sided, Bonferroni-correction was applied for p-values < 0.05)

Supplementary Notes 3: RT modulation in non-ICM trials

To rule out a hypothetical bias in reaction times (RTs) due to the pure timing of visual stimulus progression and ICM (but not the physiological impact of ICM-evoked spikes) and to quantify the level of variability within the data, we assessed the hypothetical dependence of RT delay on the actual ICM application times. We performed a bootstrapping procedure by randomly pairing the actual ICM application times with trials and corresponding RTs without ICM. Then, we calculated the RT modulation as a function of γ-phases. Then, the RT modulation as a function of γ-phases was calculated. This procedure was repeated 5000 times, resulting in a distribution of RT-modulations as a function of γ-phases. Contrary to a hypothetical bias, the median RT modulation in trials without ICMs exhibited minimal variation across γ-phases (see Supplementary Fig 4, median displayed in red, 95 % CI highlighted in gray). Additionally, the curve representing trials with ICM application exceeded the 95% confidence interval based on non-ICM trials and corresponding RTs in both animals (Supplementary Fig.4, black curve). These results indicate that the pronounced behavioral effects we observed are indeed attributable to the ICM application rather than the trial time they were applied.

Supplementary Notes 4: Effect of ICMs on γ-oscillations

To assess potential distortions of phase progressions of the ongoing γ-oscillations in V4 by ICM-evoked spikes, we analyzed the γ-phase progression in two distinct 15 ms periods. During the first period (5-20 ms after the ICM-pulse, Supplementary Fig. 6A, red bars), ICM-evoked spikes arrived in V4, while the second period (-10-5 ms to the ICM-pulse, Supplementary Fig. 6A, blue bars) served as a reference. Statistical tests indicate no significant differences between the distributions of γ-phase progressions for both 15 ms periods in both animals (monkey B: median 5-20 ms: 387.3°, median -10-5 ms: 385.7°, $n = 2311$, $p = 0.21343$, $z = 1.2442$; monkey T: median 5-20 ms: 372.3°, median -10-5 ms: 373.0°, $n = 3389$, $p = 0.1245$, $z = 1.536$; both Wilcoxon signed-rank tests, two-sided).

The very similar distributions of phase progressions indicate that the ongoing γ -oscillations in V4 are not consistently affected by ICMs in terms of phase shift or phase reset. However, this does

not rule out differential effects of ICMs, depending on the γ-phase at which ICM evoked spikes arrived in V4. To investigate this, we sorted the phase progression values for the 5 - 20 ms period based on the V4 γ-phase 9.2 ms after the ICM-pulse (Supplementary Fig. 6B). We then performed a permutation test to identify significant deviations from the expectation. For this, we randomly paired the γ-phases with phase-progression values (10,000 times) from the period 5 – 20 ms after ICM. We found no consistent effect across animals. For monkey T, neither the minimum nor the maximum showed a significant difference from expectation. For monkey B, the amplitude of the maximum was not significant after the Bonferroni correction ($p = 0.0592$, bootstrapped hypothesis testing, one-sided), while the minimum differed significantly from expectation even after the Bonferroni correction ($p = 0.0102$, bootstrapped hypothesis testing, one-sided). However, the effect is small since the minimum (7.5° decrease) corresponds to a decrease in phase progression of only 2 % from the median phase progression in a 15 ms period.

Supplementary Figures

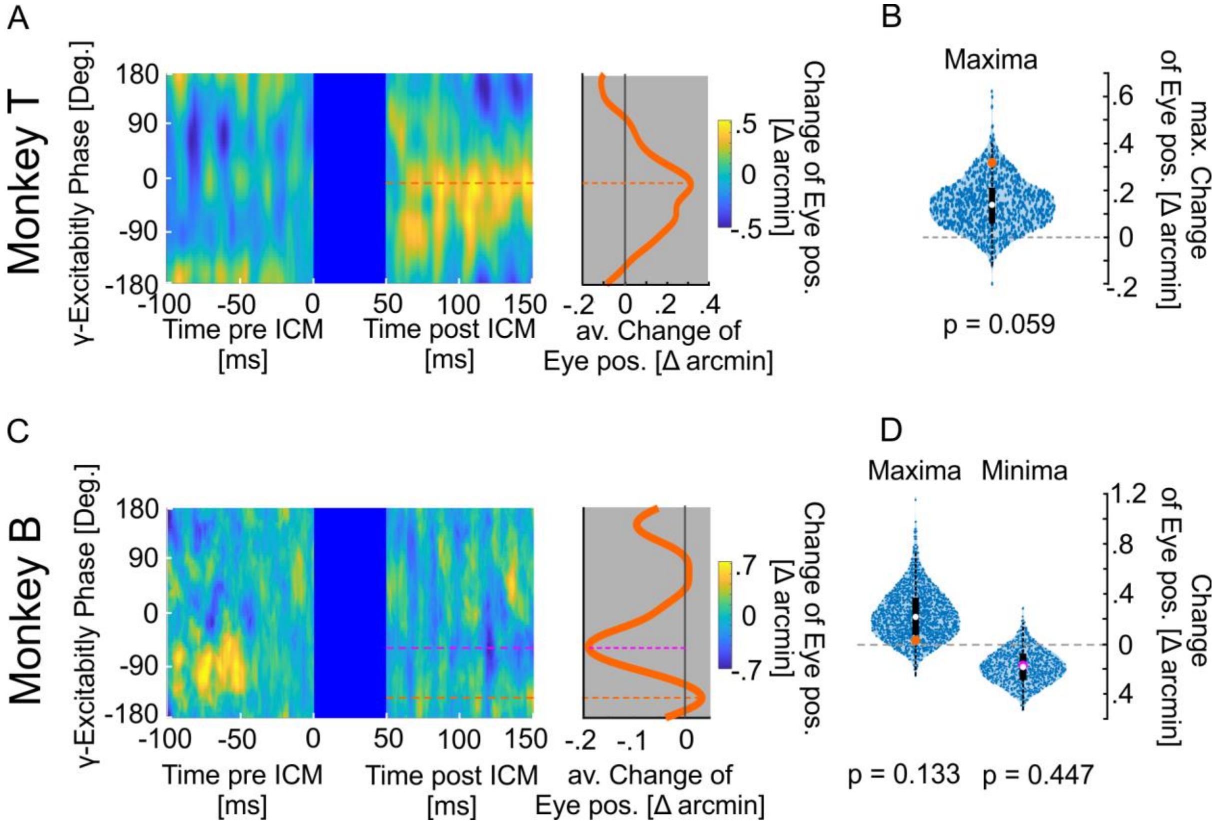

Supplementary Figure 1: ***Effects of ICMs on Eye positions***. ***A*** *The heatmap illustrates the difference between the average changes in the direction of gaze during trials with ICM and the average direction of gaze across days)n = 20) during trials without ICMs for monkey T, as a function of the V4 γ-excitability phase at 9.2 ms after the ICM pulse. Changes were calculated for ICM and noICM trials with respect to the average gaze position of the reference period 0 – 50 ms (shown in blue). The heatmap includes the differences between ICM and nonICM trials also for the 100 ms period preceding ICMs, facilitating comparisons between intervals unaffected by ICM and those potentially influenced by its effects on the direction of gaze. The red curve in the right panel depicts the average (50 to 150 ms) deviation between ICM and nonICM gaze positions across all phases. The vertical red dashed lines in the heatmap and in the panel on the right indicate the γ-phase associated with the largest difference between ICM and nonICM gaze position changes (0.32 arcmin at -7.5°).* ***B*** *Analogue to the maximum of average deviations across phases in the right panel of A (horizontal red dashed line) for ICM data, the violin plot depicts the distribution of average values taken at the maximum of each iteration (dark blue dots) of shuffle controls (n = 1000). The position of each maximum within the phase dimension was not restricted. The red dot is the maximum average deviation between ICM and nonICM data as in A (horizontal red dashed line, 0.32 arcmin). There was no significant effect of ICMs on the direction of gaze for monkey T, as the likelihood of observing the average change in eye position from ICM trials is p = 0.059 (bootstrapped hypothesis testing, one-sided).* ***C*** *Same as in A, but for monkey B (n = 16 sessions). There was no substantial peak in the difference of average gaze position changes (50 to 150 ms) between ICM and nonICM trials, but a more pronounced trough. Therefore, the maximum (red dashed lines) and minimum (magenta dashed lines) of the curve were tested against the distributions of nonICM gaze position changes in* ***D.*** *Same as in B, but for monkey B and for maximum (0.03 arcmin at -142.5°, red dot) and minimum (-0.19 arcmin at -57.5°, magenta dot) against the respective distribution of maxima and minima derived from nonICM eye position changes (n = 1000, p = 0.133 and p = 0.44, respectively, bootstrapped hypothesis testing, one-sided.). Source data are provided as a Source Data file.*

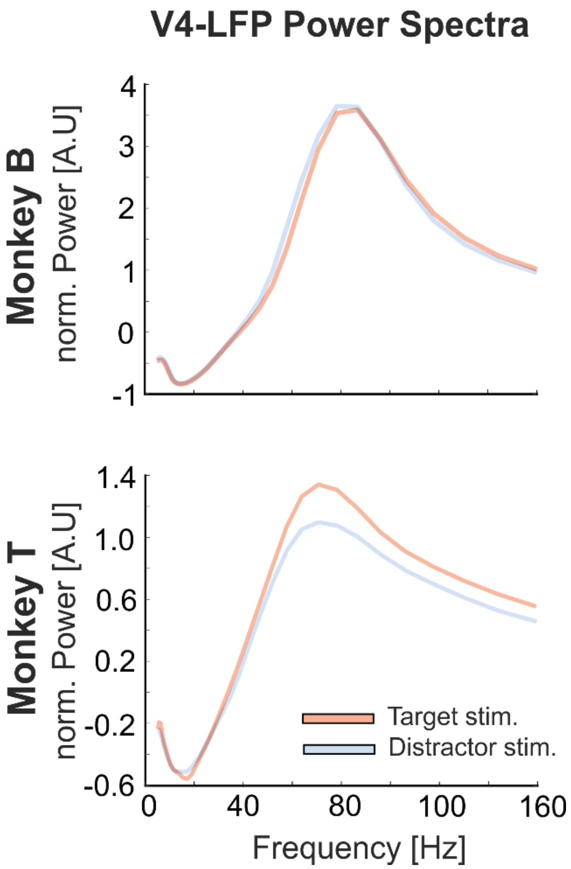

Supplementary Figure 2: ***Average power spectra of task conditions***. *Mean power spectra of V4 LFP of monkey B (top, n = 16 sessions) and monkey T (bottom, n = 20 sessions) during the conditions used to investigate γ-phase dependent effects of ICMs on RTs. These conditions contain two stimuli presented in the V4 RFs and one of them in RF of the V2 site receiving ICMs. Attention is focused on either one of the two stimuli. The red graphs represent average power spectra when the ICM-application site in V2 responded to a cued stimulus (Target stim). Blue graphs represent average power spectra when the ICM application site represented an uncued stimulus (Distractor stim.). Source data are provided as a Source Data file.*

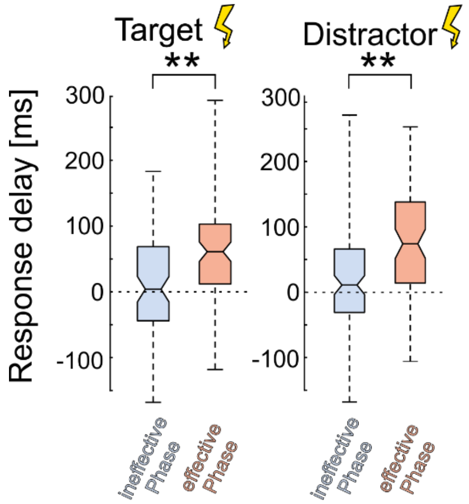

Supplementary Figure 3: ***Comparison of ICM effect on Response Times (RT) during different task conditions.*** *The Effect of ICMs on RTs during the effective (red) and ineffective (blue) phase ranges of the V4 γ-cycle. RTs are displayed as difference from the average RT of trials without ICM application. RTs from trials in which the ICM target site processed the relevant stimulus are shown on the left (Target, median ineffective = 4 ms, n = 85 trials, median effective = 61ms, n = 94 trials, p = 0.00019087, z = 3.7308, Wilcoxon rank-sum test, two-sided), and trials where the same V2 neurons processed an irrelevant stimulus are shown on the right (Distractor, median ineffective = 11 ms, n = 111 trials, median effective = 74 ms, n = 75 trials, $p = 3.5389*10^{-6}$, z = 4.6368, Wilcoxon rank-sum test, two-sided), respectively. Boxplots show the median (center line); notches indicate a confidence interval around the median, calculated as median ± 1.57 × IQR / √n. Box edges represent the interquartile range (IQR), and whiskers extend to the minimum and maximum data values. **-indicates high significance at $p < 0.01$. Source data are provided as a Source Data file.*

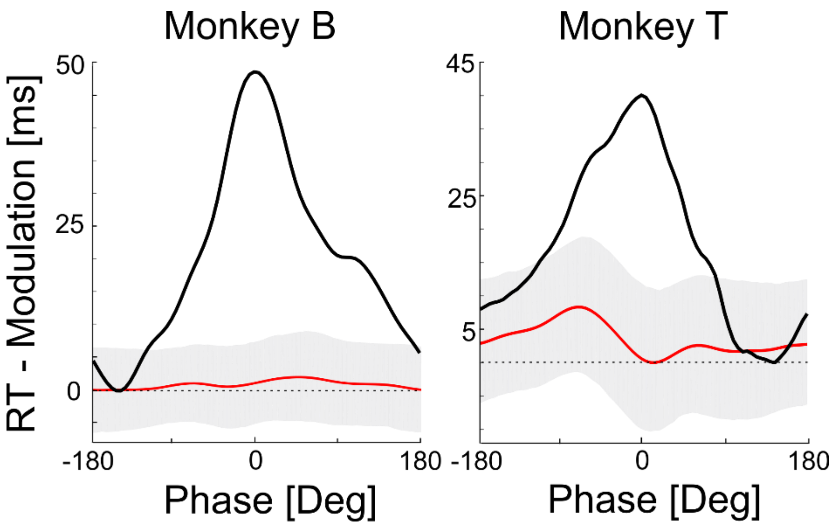

Supplementary Figure 4*: γ-phase dependent modulation of RTs from trials with and without ICM application.* *The red graphs represent the median RT-modulation (monkey B: n = 16 sessions, monkey T: n = 19 sessions) based on trials without ICM application as a function of the V4 γ-phases. The gray highlighted areas indicate the 95 % confidence interval. The black lines show the RT-modulation of ICM data for comparison. Source data are provided as a Source Data file.*

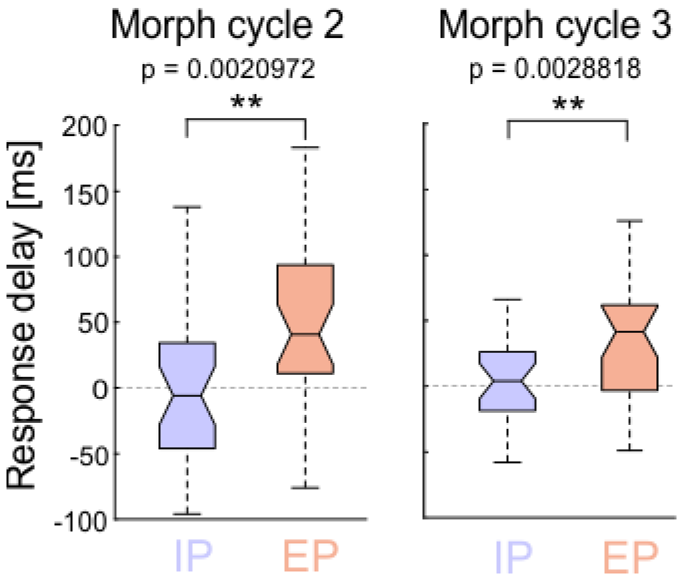

Supplementary Figure 5: Comparison of the RT-delay during morph cycles two and three (MC 2 and 3) for the ineffective (IP, blue) and effective (EP, red) phase ranges of both animals. ***The left panel*** *shows the distribution of median RT-delays with respect to RTs of nonICM trials across days for the EPs and IPs of both animals during MC 2. The sessions' median RTs of both animals were pooled since the separation of data into two groups (response for MCs 2 and 3) decreased the number of sessions included in the analysis (minimum of 5 entries required for EP and IP). The difference between median RTs following ICMs in the EP (median = 40.8 ms) was significantly different from median RTs following ICMs during the IP (median = -6 ms, n = 32, p = 0.0020972, z = 3.0761, Wilcoxon signed-rank test, two-sided).* ***The right panel*** *shows the same for MC 3, with significant differences between RTs following ICMs in the EP (median = 41.3 ms) and the IP (median = 3.8 ms, n = 28, p = 0.0028818, z = 2.9801, Wilcoxon signed-rank test, two-sided).*
Neither the RT-delays following ICM pulses during the EP in MC2 and MC3 were significantly different (p = 0.26962, z = 1.1039), nor were the differences between RTs following ICM pulses during the IPs (p = 0.55829, z = 0.58538, both Wilcoxon rank-sum test, two-sided).
*The boxplots show the medians and interquartile ranges (IQR); whiskers extend to data points within 1.5×IQR. ** indicates significance at p < 0.01. Source data are provided as a Source Data file.*

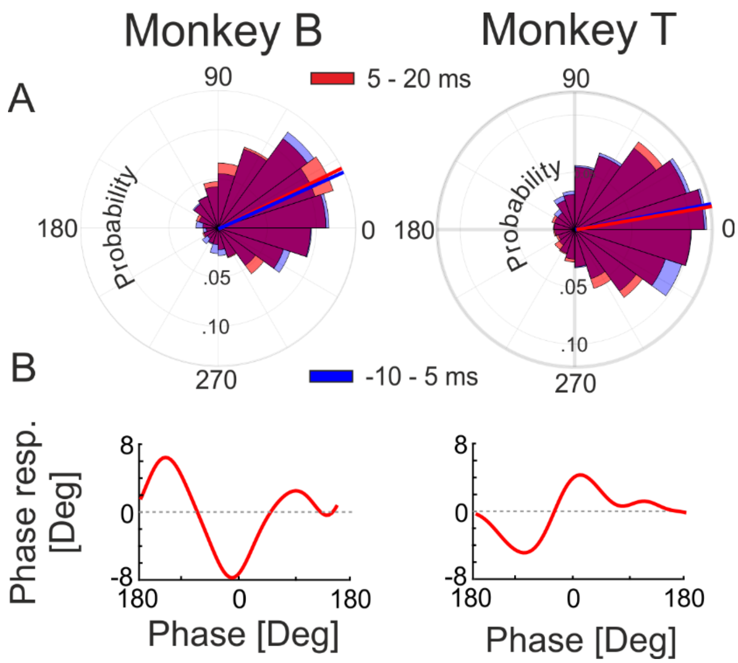

Supplementary Figure 6: ***Effect of ICM on γ-oscillations. A*** *The polar histograms depict the distribution of γ-phase progression values within two distinct 15 ms periods of individual data segments for both animals. The bars for the period 5 – 20 ms (with respect to the ICM-pulse) are shown in red. For comparison, the phase progression distribution for the period -10 to 5 ms are presented, which precedes the arrival of ICM-evoked spikes. The red and blue lines depict the median phases for the corresponding distributions.* ***B*** *γ -phase response curve for the period 5 – 20 ms (red in A) plotted as a function of the γ-excitability -phase 9.2 ms after ICM. The values indicate the difference with respect to the median phase progression in the -10 to 5 ms period (blue in A). Source data are provided as a Source Data file.*

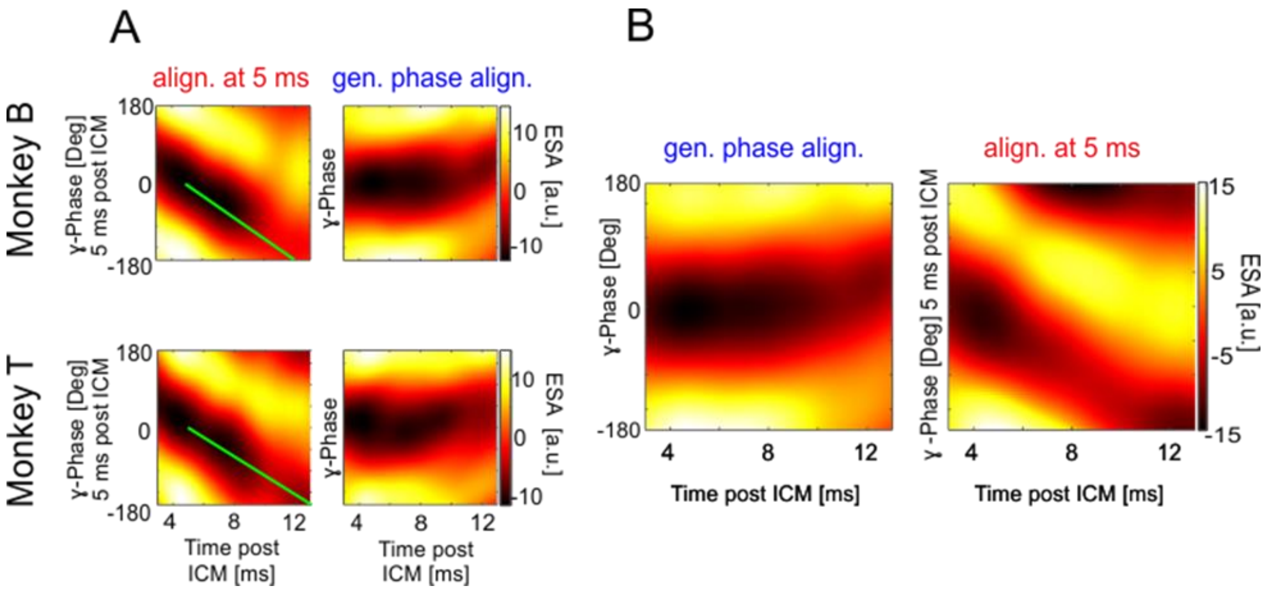

Supplementary Figure 7: ***Illustration of the harmonization of γ-oscillation-dependent ESA time-courses between animals.*** *To pool the ESA across animals with different γ-frequencies, their differences in γ-phase progression had to be removed.* ***A*** *Average ESA-time course following ICM-application depending on γ-phase for monkey B (top left) and monkey T (bottom left). The individual ESA data segments were sorted according to their LFP γ-phase at 5 ms after the ICM-pulse (aligned at 5 ms), and a moving average in the phase dimension was calculated for each time bin using a Gaussian kernel (σ = 30°, window: 180° steps of 5°, see Methods). Note that the LFP γ-oscillations have their trough at 0°, while the antiphasic excitability cycle peaks at 0°. The heat maps illustrate the strong intrinsic LFP γ-phase dependence of spiking activity, showing that the highest activity is concentrated at the trough of the LFP γ-oscillation (at 5 ms). Over time, the peak of spiking activity systematically shifts through the gamma phases, which is illustrated by the green bars. The right panels depict the same data but with a different sorting approach. The ESA values were sorted for each time bin according to the forecasted γ-phases (generally phase-aligned). The horizontal alignment of both animals' high and low ESA values (stripes) confirms the accurate prediction of γ-phases following the estimated phase at 5 ms.* ***B*** *The left panel displays the phase-aligned ESA (each time bin) following ICMs, averaged across both animals. The right panel shows the time course of the average ESA after reconstruction with the average frequency of both animals (see Methods).*

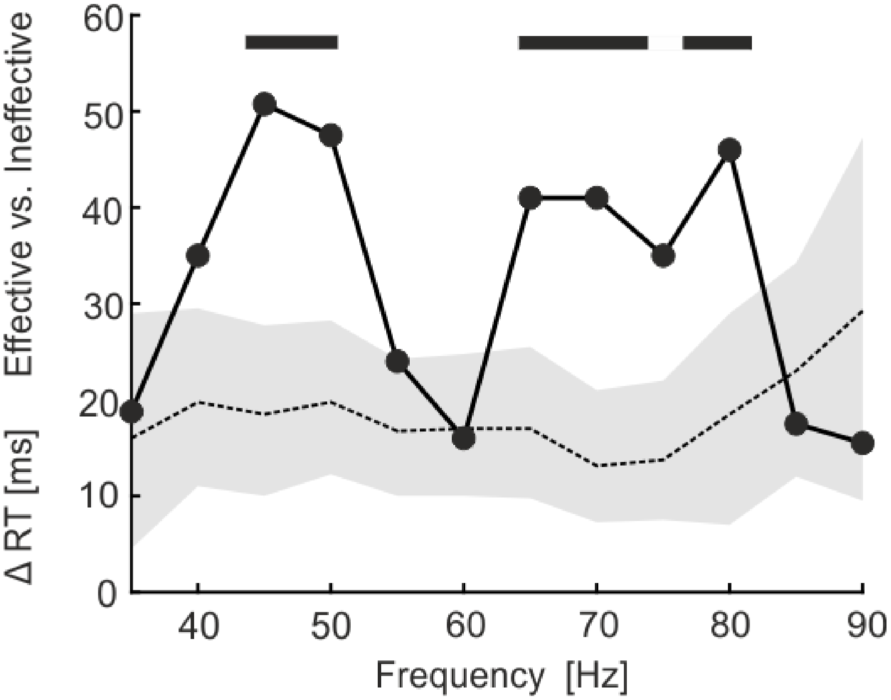

Supplementary Figure 8: ***Comparison of γ-phase dependent effects of ICM on RT-modulation for different frequency bands****. For this the instantaneous frequency (see methods) within a frequency range of 20 to 110 Hz at the time around ICMs (-5 to 15 ms) was used. RTs associated with frequencies falling into a window of 20 Hz width (5 Hz steps) were taken and based on these cases and associated γ-phases, the RT modulation as a function of the γ-excitability phase calculated for each frequency range of each session of each animal. The median RT-modulation curve of each animal delivered the frequency range and animal specific effective and ineffective phase ranges (± 45° around maximum and minimum respectively). All RTs associates with either the ineffective or effective phase ranges were pooled across days and animals. The black trace with black dots shows the spread between median RTs of the effective and ineffective phase ranges for each frequency range of 20 Hz width. Since for each frequency range the effective and ineffective phase ranges were defined based on the animal specific median RT-modulation curve, all values are well above 0 ms. Shuffle controls were performed to test for significance, by randomly aligning actual phases and RTs for each frequency range and session of both animals. The following procedures mirrored that of the ICM data, resulting in median RT-modulation curves based on random phase to RT relations. These curves delivered the effective and ineffective phase ranges and RTs falling into the animal specific phase ranges were pooled across sessions and animals. The area highlighted in gray represents the first and third quartile of the shuffle control (n = 5,000) spreads between median RTs of the effective and ineffective phase ranges. The black dashed line indicates the median of this shuffle control distribution. The horizontal black bars on top indicate differences between ICM spreads and the shuffle control at $p < 0.05$. The number of trials and associated RTs to calculate the RT spread were: $n_{effective} = 51$, $n_{ineffective} = 51$ (center 35 Hz), $n_{effective} = 55$, $n_{ineffective} = 64$ (center 40 Hz), $n_{effective} = 78$, $n_{ineffective} = 83$ (center 45 Hz), $n_{effective} = 85$, $n_{ineffective} = 85$ (center 50 Hz), $n_{effective} = 95$, $n_{ineffective} = 98$ (center 55 Hz), $n_{effective} = 101$, $n_{ineffective} = 92$ (center 60 Hz), $n_{effective} = 94$, $n_{ineffective} = 72$ (center 65 Hz), $n_{effective} = 88$, $n_{ineffective} = 96$ (center 70 Hz), $n_{effective} = 73$, $n_{ineffective} = 96$ (center 75 Hz), $n_{effective} = 51$, $n_{ineffective} = 75$ (center 80 Hz), $n_{effective} = 35$, $n_{ineffective} = 52$ (center 85 Hz), $n_{effective} = 26$, $n_{ineffective} = 33$ (center 90 Hz)*

Note, that although the filter covered the β- and high γ-bands, these frequencies occurred to rarely to calculate RT-modulation curves. Source data are provided as a Source Data file.

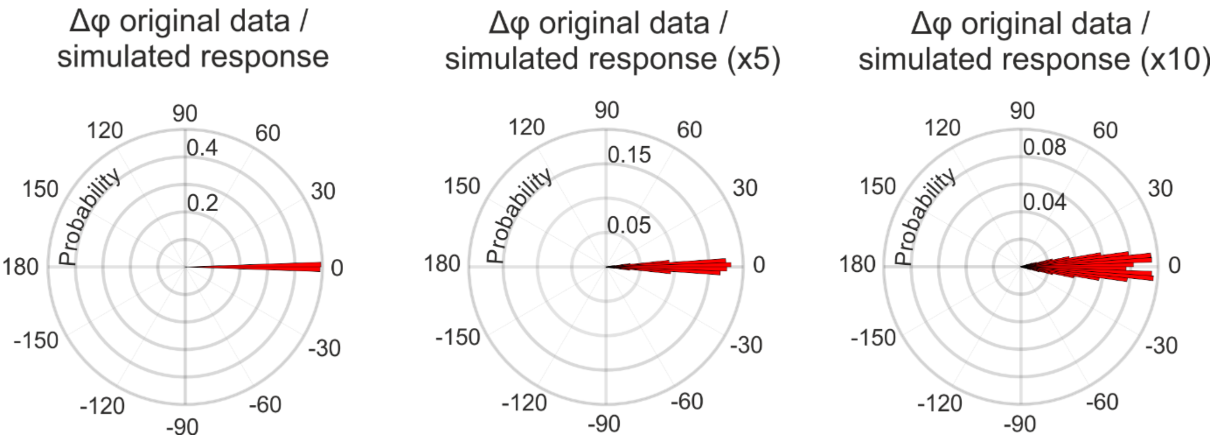

Supplementary Figure 9: ***Effect of ICM-evoked response on phase estimation.*** *To test whether ICM-evoked responses could bias γ-phase estimation, we compared phase estimates from unperturbed data segments (filtered and Hilbert-transformed) with those from the same segments after adding the maximum average ICM-evoked response observed in the V4 LFP (Fig. 3B, top panel). This data with simulated responses was processed identically (see methods section for details). The* ***Left panel*** *shows the distribution of the differences between γ-phase estimates in the data with and without simulated response (n = 8,502). The median absolute difference was 0.56°, with 95% of γ-phase differences below 1.47°. The* ***Middle panel*** *shows the same analysis but with simulated responses five times larger than the actual maximum average ICM-evoked response observed in the V4 LFP. Even under this exaggerated condition, phase distortions remained minor (median absolute difference: 2.7°, 95% < 7.7°). The* ***Right panel*** *shows the same as above but with a simulated response ten times the actually observed maximum response. Despite this extreme manipulation, the disturbance of the γ-phase estimation remained very limited (median absolute difference: 5.5°, 95% < 15.0°).*

These analyses show that the phase-dependent effects of ICMs on the ESA and the LFP in V4 (Fig. 3) cannot be explained by disturbances of γ-phase estimation. ESA and LFP responses occurred within a narrow effective phase range spanning about 90° around the most effective phase. Inducing such a bias in a set of actually randomly distributed phase values would need to shift phase values substantially and systematically by up to ±135° to change the uniform distribution into an unimodal distribution mainly within the effective phase range. However, even responses ten times stronger than the maximum actually observed only induced an average phase difference of 5.5°, while the actual maximum response caused changes of just 0.56° on average. These phase changes are far too small compared to the large phase shifts that would be necessary to explain the observed strong concentration of responses within the effective phase range with a bias in the phase estimates due to the ICM-evoked responses in the LFP.

.

	Monkey B			Monkey T		
	ICM	No ICM.	p- and z-value	ICM	No ICM.	p- and z-value
Misses.	11.0 %	6.7 %	0.0093 / 2.96	6.9 %	4.3 %	0.035 / 2.528
False Alarms	8.6 %	6.7 %	0.149 / 1.4438	9.6 %	14.0 %	$2.93*10^{-5}$ / 4.422
Eye Errors	10.2 %	12.7 %	0.149 /1.444	14.4%	16.2 %	0.06 / 2.303

Supplementary Table 1: *Comparison of the frequency of behavioral errors between trials with ICM versus without ICM (No ICM) and equal visual stimulation. The p- and z-values are the results of Wilcoxon signed-rank tests (two-sided); p-values < 0.05 were corrected for multi-comparison using Bonferroni correction; monkey B: n = 17; monkey T: n = 40. Source data are provided as a Source Data file.*
