# Supplementary material for: Gamma-band synchronization between neurons in the visual cortex is causal for effective information processing and behavior
Source: Nat Commun. 2025 Aug 11;16:7380. doi: 10.1038/s41467-025-62732-8 (PMC12340064; doi:10.1038/s41467-025-62732-8)
Supplement: Supplementary file 2 — Reporting Summary [file 41467_2025_62732_MOESM2_ESM.pdf]

nature portfolio

Corresponding author(s): Eric Drebitz, Andreas K. Kreiter

Last updated by author(s): Jul 9, 2025

Reporting Summary

Nature Portfolio wishes to improve the reproducibility of the work that we publish. This form provides structure for consistency and transparency in reporting. For further information on Nature Portfolio policies, see our Editorial Policies and the Editorial Policy Checklist.

Statistics

For all statistical analyses, confirm that the following items are present in the figure legend, table legend, main text, or Methods section.

n/a	Confirmed	
☐	☒	The exact sample size (*n*) for each experimental group/condition, given as a discrete number and unit of measurement
☐	☒	A statement on whether measurements were taken from distinct samples or whether the same sample was measured repeatedly
☐	☒	The statistical test(s) used AND whether they are one- or two-sided<br>*Only common tests should be described solely by name; describe more complex techniques in the Methods section.*
☐	☒	A description of all covariates tested
☐	☒	A description of any assumptions or corrections, such as tests of normality and adjustment for multiple comparisons
☐	☒	A full description of the statistical parameters including central tendency (e.g. means) or other basic estimates (e.g. regression coefficient) AND variation (e.g. standard deviation) or associated estimates of uncertainty (e.g. confidence intervals)
☐	☒	For null hypothesis testing, the test statistic (e.g. *F*, *t*, *r*) with confidence intervals, effect sizes, degrees of freedom and *P* value noted<br>*Give P values as exact values whenever suitable.*
☒	☐	For Bayesian analysis, information on the choice of priors and Markov chain Monte Carlo settings
☐	☒	For hierarchical and complex designs, identification of the appropriate level for tests and full reporting of outcomes
☒	☐	Estimates of effect sizes (e.g. Cohen's *d*, Pearson's *r*), indicating how they were calculated

Our web collection on statistics for biologists contains articles on many of the points above.

Software and code

Policy information about availability of computer code

Data collection	Neuronal data was collected with the commercially available software "MC Rack", version 4.6.2., (release date: 2015-02-12), provided by Multi-Channel Systems (MCS) GmbH, Reutlingen, Germany.
Data analysis	The data was analyzed using custom MATLAB scripts (versions R2016b and R2020a, MathWorks, Natick, MA, USA). A comprehensive and detailed description of all analyses is provided in the methods section. Electrical artifact removal was conducted following the method published in "Journal for Neuroscience Methods" (Drebitz et al., 2020, doi: 10.1016/j.jneumeth.2019.108549). Circular statistics used in the supplementary information were performed using the open-source Matlab toolbox v.1.21.0.0 (downloaded 06/14/2017) "CircStat: a Matlab toolbox for circular statistics", as referenced. The analyses do not involve custom algorithms or software, central to the research but are not yet described in published literature. However, the custom-made scripts can be provided upon reasonable request.

For manuscripts utilizing custom algorithms or software that are central to the research but not yet described in published literature, software must be made available to editors and reviewers. We strongly encourage code deposition in a community repository (e.g. GitHub). See the Nature Portfolio guidelines for submitting code & software for further information.

Data

Policy information about availability of data

All manuscripts must include a data availability statement. This statement should provide the following information, where applicable:

- Accession codes, unique identifiers, or web links for publicly available datasets
- A description of any restrictions on data availability
- For clinical datasets or third party data, please ensure that the statement adheres to our policy

The data supporting this study are available from the corresponding author (drebitz@brain.uni-bremen.de) upon reasonable request due to their size, complexity, and context-dependency. Source data are provided with this paper.

Research involving human participants, their data, or biological material

Policy information about studies with human participants or human data. See also policy information about sex, gender (identity/presentation), and sexual orientation and race, ethnicity and racism.

Reporting on sex and gender	*Use the terms sex (biological attribute) and gender (shaped by social and cultural circumstances) carefully in order to avoid confusing both terms. Indicate if findings apply to only one sex or gender; describe whether sex and gender were considered in study design; whether sex and/or gender was determined based on self-reporting or assigned and methods used. Provide in the source data disaggregated sex and gender data, where this information has been collected, and if consent has been obtained for sharing of individual-level data; provide overall numbers in this Reporting Summary. Please state if this information has not been collected. Report sex- and gender-based analyses where performed, justify reasons for lack of sex- and gender-based analysis.*
Reporting on race, ethnicity, or other socially relevant groupings	*Please specify the socially constructed or socially relevant categorization variable(s) used in your manuscript and explain why they were used. Please note that such variables should not be used as proxies for other socially constructed/relevant variables (for example, race or ethnicity should not be used as a proxy for socioeconomic status). Provide clear definitions of the relevant terms used, how they were provided (by the participants/respondents, the researchers, or third parties), and the method(s) used to classify people into the different categories (e.g. self-report, census or administrative data, social media data, etc.) Please provide details about how you controlled for confounding variables in your analyses.*
Population characteristics	*Describe the covariate-relevant population characteristics of the human research participants (e.g. age, genotypic information, past and current diagnosis and treatment categories). If you filled out the behavioural & social sciences study design questions and have nothing to add here, write "See above."*
Recruitment	*Describe how participants were recruited. Outline any potential self-selection bias or other biases that may be present and how these are likely to impact results.*
Ethics oversight	*Identify the organization(s) that approved the study protocol.*

Note that full information on the approval of the study protocol must also be provided in the manuscript.

Field-specific reporting

Please select the one below that is the best fit for your research. If you are not sure, read the appropriate sections before making your selection.

☒ Life sciences ☐ Behavioural & social sciences ☐ Ecological, evolutionary & environmental sciences

For a reference copy of the document with all sections, see nature.com/documents/nr-reporting-summary-flat.pdf

Life sciences study design

All studies must disclose on these points even when the disclosure is negative.

Sample size	Behavioral effects were computed based on recordings from 17 and 40 sites in area V4 of two macaque monkeys (Macaca mulatta), respectively. After data exclusions, these sessions yielded 9762 and 7631 data segments, respectively, with single pulses of intracortical microstimulation in V2 for subsequent analyses of spiking activity and the local field potential. Statistical parameters like variances, effect sizes, and noise levels were unknown beforehand. Consequently, no a priori power calculations were conducted. The number of animals, V4 recording sites, and data segments integrated into the analyses adheres to typical practices within the field.
Data exclusions	The initial assessment of the impact of intra-cortical microstimulation (ICMs) on the proportion of missed responses encompassed all recording sessions and sites. To make sure that only measurements of successfully performed experiments were used, the subsequent analyses were constrained to sessions and sites meeting two criteria: first, a greater proportion of missed responses in trials with ICM compared to those without ICM (to ensure successful and effective microstimulation); second, an increase in gamma-band power by at least 70% during stimulus presentation relative to the baseline period to ensure that the relevant stimulus induces gamma-band activity and allows for meaningful phase estimates. This resulted in n = 60 and n = 102 sites for the two monkeys. For the comparison of reaction times, we required, in addition, a minimum number of 15 trials without ICM application in which the animals executed the task correctly to ensure a

	meaningful estimation of the median reaction times as well as an electrode tip location in granular layers (resulting in n = 16 and n = 20 sites, respectively). The methods section under "Data Selection" details these general exclusion and further analysis-specific criteria.
Replication	None. A repetition would require the training and data recording in area V4 of additional monkeys, which would raise ethical concerns and deviate from the field's standard practices.
Randomization	Both monkeys were exposed to pseudo-randomized behavioral conditions, rendering it impossible to foresee the reappearance of the target shape and whether ICM was applied. The recordings within the V4 region of interest resulted in a random sampling of neuronal activity, as sites were not chosen based on physiological response characteristics like stimulus tuning. ICM was randomly applied to the target region in V2 during the latter half of each morphing cycle of the stimuli. This approach ensured that the timing of the ICM application could not be predicted.
Blinding	During data collection, the computer randomly chose all relevant parameters pseudo-randomly just before each behavioral trial started. Within data analysis, the selection criteria were implemented using automated MATLAB scripts, eliminating the need and the possibility for manual data selection. The analysis pipeline was entirely automated and devoid of any user input.

Reporting for specific materials, systems and methods

We require information from authors about some types of materials, experimental systems and methods used in many studies. Here, indicate whether each material, system or method listed is relevant to your study. If you are not sure if a list item applies to your research, read the appropriate section before selecting a response.

Materials & experimental systems

n/a	Involved in the study	
☒	☐	Antibodies
☒	☐	Eukaryotic cell lines
☒	☐	Palaeontology and archaeology
☐	☒	Animals and other organisms
☒	☐	Clinical data
☒	☐	Dual use research of concern
☒	☐	Plants

Methods

n/a	Involved in the study	
☒	☐	ChIP-seq
☒	☐	Flow cytometry
☒	☐	MRI-based neuroimaging

Animals and other research organisms

Policy information about studies involving animals; ARRIVE guidelines recommended for reporting animal research, and Sex and Gender in Research

Laboratory animals	The study involved two adult male macaque monkeys (Macaca mulatta), aged 14 and 15.
Wild animals	The study does not involve wild animals.
Reporting on sex	Sex-based analyses are not part of the study. The research focuses on whether a proposed fundamental mechanism of signal and information routing exists or does not exist. Such fundamental neuronal mechanisms are presumed to be consistent between sexes. Investigations on potential, most likely limited, quantitative, sex-dependent differences of parameters of this mechanism would require a different study with a much higher number of animals.
Field-collected samples	No field-collected samples.
Ethics oversight	All procedures and animal care were endorsed by local authorities (Senator für Gesundheit, Bremen, Germany). Additionally, the experimental protocols adhered to the German Animal Welfare Act (TierSchG) and followed the guidelines outlined in the European Union's directive (2010/63/EU) for the care and utilization of laboratory animals.

Note that full information on the approval of the study protocol must also be provided in the manuscript.
